# Clinical Cases

**Published:** 1899-04

**Authors:** C. C. Howard

**Affiliations:** New York City


### CLINICAL CASES.

C. C. Howard, M. D., New York City.

The first case I wish to present to you is that of an old lady about sixty years of age. I saw her first about ten years ago after the loss of her daughter. She suffered extremely from grief. She had gout for a long time, was very melancholy, and would weep at the slightest provocation. She had dreams about conversations with the dead. The gout was in the form of general soreness all over the body, and she was extremely sensitive to touch. The slightest change of the weather would make her worse. Appetite poor. Complained of everything smelling bad to her. Over-sensitiveness of taste. Mental restlessness. I prescribed for her, and she would be relieved for a time, then would come a change in the weather, and the old condition would come back again. I changed the potency, and she would improve and then go back again. I used Sulph. and Kali-carb. She would be startled on closing of door. Vertigo on one side of the head. For different conditions she had Belladonna, Arnica, and Lycopodium. She finally decided to go to Europe, which she did, and spent most of the time in France. She came back much improved in every way. Gout gone, excessive sensitiveness of smell gone. She looked in prime condition. I did not see her for several months. When I saw her she had become very thin, she had great dryness of the mouth and intense thirst. Complained of constant desire to urinate. Skin was very dry and of scaly appearance. I examined the urine and found 6½ to 7 per cent. sugar. What she had been doing in France I could not find out. This state continued, and she grew very much worse. Phosphoric-acid helped her for quite a time. No remedy helped her for any length of time. Later she complained of intense itching all over the abdomen, genitals, etc. She had not told me about it before because she was afraid I might want to look at it. She also had some boils. Besides this she complained of great weakness, and lost all strength from her extremities. The knee-jerk had gone. I gave her one dose of Kali-brom. She improved in a week or ten days, very gradually. First, I noticed that she was better able to walk, less urine, less thirst, looked less haggard, and in three months' time there was less than 3½ per cent. of sugar. I think she had in all three doses. She insisted on going into her daughter's room, which had never been touched since her death. She developed pneumonia, lived three days, and died. The diminution of sugar in the urine was remarkable, however, and the itching disappeared entirely and the appetite became normal.

Case 2.—An old German, fifty or sixty years of age, had several attacks of gout and renal calculi. He drank three or four bottles of wine in a day, and would top off with a drink of whisky and a couple of other things. He had gout in his toe. That disappeared suddenly; he lost his knee-jerk, passing a good deal of water. I examined his urine and found 2½ per cent. of sugar in it. He had pimples on his face and nose. He had had a good deal of business anxiety. One dose of Kali-brom. completely cured that man, and he has had no return of his old condition. No return of the sugar in the urine.

Case 3. An old lady, sixty years of age. She suffered some from rheumatism and a gouty condition, but was in fair health. She lost her daughter, and about a year afterwards her husband dropped dead of apoplexy—died very suddenly. She lost a son six months afterward of pneumonia, and another son about six months before that of cancer. I saw her about the time of the son's death, and she had all the symptoms of diabetes. Excessive amount of urine, falling out of hair, excessively dry skin. I was called to see her especially in regard to her leg, which had turned black and was the seat of severe pain. I prescribed for her, and told her if collateral circulation would take place she would get well, but if not, gangrene would result. I gave her Sulphur, the condition left her, and she gained entire use of her limb. She had the same bad condition of the skin as in Case 1, and was afraid I would want to look at it. I prescribed Kali-brom., and in about four weeks the sugar went down from 5 per cent. to less than 2 per cent. Now during the last five years that she has been under my care the sugar has not gone above 2 per cent, and there is no sugar in the urine now. I have let her eat most anything she wanted except food with too much starch in it. I advised her to abstain from tea or coffee.

In these three cases the principal elements seem to be the history of mental shock, the eruptions on the skin, and the multiple anxieties.

### Discussion.

Dr. Pease—Was there any history in these cases of the former abuse of Bromide of Potassium?

Dr. Howard—I have no reason to suppose that there was any such abuse.

Dr. Patch.—Was any particular diet used in the other two cases?

Dr. Howard—No, sir. I did try to put the woman on a milk diet, but it did no good. I advised them all to refrain from eating too much food with starch in it.

Dr. Kennedy—There was considerable trouble with the nervous system in these cases, was there not, with great cares?

Dr. Howard—Yes, there was considerable mental anxiety and cares, in one from loss of members of the family, in the other from business affairs.

Dr. Kennedy—The excellent results from the use of Kali-brom. are remarkable.

